# Functional Characterization of a New GH107 Endo-α-(1,4)-Fucoidanase from the Marine Bacterium *Formosa haliotis*

**DOI:** 10.3390/md18110562

**Published:** 2020-11-17

**Authors:** Marlene Vuillemin, Artem S. Silchenko, Hang Thi Thuy Cao, Maxim S. Kokoulin, Vo Thi Dieu Trang, Jesper Holck, Svetlana P. Ermakova, Anne S. Meyer, Maria Dalgaard Mikkelsen

**Affiliations:** 1 Protein Chemistry and Enzyme Technology Section, DTU Bioengineering, Department of Biotechnology and Biomedicine, Technical University of Denmark, 2800 Kgs Lyngby, Denmark; mavu@dtu.dk (M.V.); tvtd@dtu.dk (V.T.D.T.); jesho@dtu.dk (J.H.); asme@dtu.dk (A.S.M.); 2 NhaTrang Institute of Technology Research and Application, Vietnam Academy of Science and Technology, 02 Hung Vuong Street, Nhatrang 650000, Vietnam; caohang.nitra@gmail.com; 3 G.B. Elyakov Pacific Institute of Bioorganic Chemistry, Far-Eastern Branch of the Russian Academy of Sciences, 159, Prospect 100-let Vladivostoku, 690022 Vladivostok, Russia; artem.silchencko@yandex.ru (A.S.S.); maxchem@mail.ru (M.S.K); ermakova@piboc.dvo.ru (S.P.E.)

**Keywords:** fucoidan, fucoidanase, *Fucus evanescens*, GH107, fuco-oligosaccharides

## Abstract

Fucoidans from brown macroalgae are sulfated fucose-rich polysaccharides, that have several beneficial biological activities, including anti-inflammatory and anti-tumor effects. Controlled enzymatic depolymerization of the fucoidan backbone can help produce homogeneous, defined fucoidan products for structure-function research and pharmaceutical uses. However, only a few endo-fucoidanases have been described. This article reports the genome-based discovery, recombinant expression in *Escherichia coli*, stabilization, and functional characterization of a new bacterial endo-α-(1,4)-fucoidanase, Fhf1, from *Formosa haliotis*. Fhf1 catalyzes the cleavage of α-(1,4)-glycosidic linkages in fucoidans built of alternating α-(1,3)-/α-(1,4)-linked l-fucopyranosyl sulfated at C2. The native Fhf1 is 1120 amino acids long and belongs to glycoside hydrolase (GH) family 107. Deletion of the signal peptide and a 470 amino acid long C-terminal stretch led to the recombinant expression of a robust, minimized enzyme, Fhf1Δ470 (71 kDa). Fhf1Δ470 has optimal activity at pH 8, 37–40 °C, can tolerate up to 500 mM NaCl, and requires the presence of divalent cations, either Ca^2+^, Mn^2+^, Zn^2+^ or Ni^2+^, for maximal activity. This new enzyme has the potential to serve the need for controlled enzymatic fucoidan depolymerization to produce bioactive sulfated fucoidan oligomers.

## 1. Introduction

Fucoidans are a family of complex, sulfated polysaccharides found primarily in the cell wall of brown marine macroalgae (Phaeophyceae). Due to the potential beneficial bioactivities of fucoidans, which make them attractive for pharmaceutical uses, these polysaccharides have gained increasing interest over the last decade. Fucoidans are mainly composed of a backbone of α-l-fucose residues and are classified into two main classes based on the stereochemistry of the fucose backbone. Class 1 fucoidans of the brown algal orders Laminariales and Ectocarpales are defined by α-(1,3) linked l-fucosyl residues; while class 2 fucoidans of the Fucales order are built from alternating repeating units of α-(1,3)/α-(1,4)-linked l-fucosyl residues [1,2]. Although fucoidans generally have a backbone built of α-l-fucosyl residues, they are highly heterogeneous and display significant structural diversity. Hence, depending on the algal source, harvesting season, and purification process, fucoidans vary in terms of the degree of sulfation, sulfation pattern, size, and degree of branching. They also vary in the extent to which other sugars, such as galactose, glucuronic acid, mannose, rhamnose, and/or xylose residues are present, as well as substitution with acetyl groups [1,3,4].

Both fucoidan polysaccharides and fucoidan oligosaccharides have been reported to display a broad array of valuable biological activities [5,6]. These effects have mainly been shown *in vitro* in different cell systems, but in some cases also *in vivo* in animal systems, including anticancer [7,8,9,10,11,12], anti-inflammatory [1,13], anticoagulant [14,15], immune-modulatory [16], and most recently, potentially protective effects against age-related macular degeneration induced vision loss [17]. Although details of the structure-function of fucoidans remain unclear, it is evident that structural integrity is critical for achieving particular effects [1,2,17]. Fucoidan extracts have already been partly commercialized as functional food ingredients or dietary supplements [5,18], but the heterogeneous nature of fucoidan is an obstacle to achieving regulatory approval for clinical pharmaceutical uses. One option for solving this dilemma is to employ enzymes that can selectively catalyze depolymerization of fucoidans to deliver well-defined product structures that exert consistent and specific bioactivity properties, such as preserved sulfation patterns depending on their origin. Certain beneficial properties of fucoidans may even increase when the molecular weight is decreased by controlled depolymerization [19,20]. 

Fucoidanases (EC 3.2.1.211 and 3.2.1.212) catalyze the depolymerization of fucoidans and sulfated fucans. These enzymes are mainly produced by marine bacteria, but also by a few fungi and some invertebrates [21,22], and are classified in CAZy families GH107 and GH168 [23]. Some other fucoidanases have been identified, but have not yet been classified into a CAZy family [24]. In the CAZy database, the family GH107 currently contains 17 fucoidanases, and only 6 of them have been functionally characterized. The GH168 family has been created in 2020 after an endo-α-(1,3)-fucanase activity was reported for the FunA protein (GenBank accession number ACI65020.1) isolated from *Wenyingzhuangia fucanilytica* [25]. Members of the GH107 and GH168 families do not share any sequence similarity. Among the characterized GH107 enzymes, Fda1 and Fda2 from the marine bacterium *Alteromonas* sp. SN-1009 was reported to cleave α-(1,3) bonds in fucoidan isolated from *Saccharina sculpera* [26]. In 2006, the first member of the GH107 family, MfFcnA, was identified in *Mariniflexile fucanivorans*, cloned and characterized in *E. coli*. The MfFcnA fucoidanase specifically catalyzes hydrolysis of α-(1,4) fucosyl bonds in class 2 fucoidans [27]. Other fucoidanases have subsequently been identified, e.g., FFA2 from *Formosa algae* KMM 3553T and FWf1 and FWf2 from *W. fucanilytica* [28]. These enzymes are all characterized as endo α-(1,4) fucoidanases that catalyze cleavage of α-(1,4)-bonds between sulfated l-fucose residues in fucoidan extracted from *Fucus evanescens* [29]. 

The first two 3D structures of fucoidanases were solved in 2018 for the previously characterized MfFcnA, and for P5AFcnA, a GH107 fucoidanase was isolated from *Psychromonas* sp. [30]. MfFcnA revealed a four-domain organization comprising a large N-terminal (α/β)_8_-barrel catalytic domain, called D1, linked to three contiguous Immunoglobulin (Ig)-like domains whose function remains unknown. P5AFcnA displays a single domain organization and comprises only the N-terminal catalytic domain, D1. The analysis of the two crystal structures showed that the architecture of the active site of GH107 fucoidanases is highly variable, which is consistent with the great structural diversity of fucoidans. 

However, the enzymes of the family GH107 remain under-investigated. Discovery and characterization of new fucoidan depolymerizing enzymes are imperative to help deepen the understanding of the function of enzymes in this family. In addition, it is important to expand the repertoire of GH107 enzymes as tools for controlled fucoidan depolymerization to attain more homogeneous molecules for bioactivity assessments and to support elucidation of the fine and complex chemical structures of fucoidans [31].

In this study, we report identifying a gene coding for a putative fucoidanase in the genome of the marine bacterium *F. haliotis*. This gene was cloned and expressed in *E. coli*, and the corresponding protein, named Fhf1, was stabilized by targeted DNA sequence truncation and then biochemically characterized. Substrate specificity and product formation were also investigated to determine the precise mode of action of the new fucoidanase. 

## 2. Results and Discussion

### 2.1. Primary Structure of Fhf1

**Figure 1:**
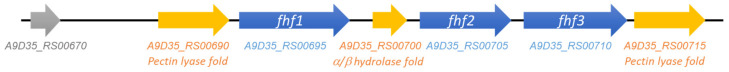
Genetic environment of fhf1 in *Formosa haliotis*. Putative fucoidanase encoding genes are colored blue, unknown open reading frames are shown in grey and yellow. Other putative protein folds (annotated from BLASTp analysis) are indicated in orange text. The locus tag number is given for each gene.

Three putative GH107 encoding genes, named *fhf1*, *fhf2*, and *fhf3*, are located close to each other in an operon in the genome sequence of the brown algae-degrading bacterium, *F. haliotis*, isolated from the gut of an abalone, *Haliotis gigantean* [32] (Figure 1). In this study, we focused on cloning and expressing *fhf1*. The primary structure of the corresponding protein (Genbank accession number WP_066217780), annotated as a T9SS type A sorting domain-containing protein in the NCBI protein database, has been investigated. 

The Fhf1 protein is 1120 amino acids long and contains a predicted 24 amino acid long N-terminal signal peptide. BLASTp analysis identified the best hit with a putative protein from *Cellulophaga* sp. L1A9, annotated as a T9SS type A sorting domain-containing protein [33]. They share 59% of identity and 73% of similarity on 99% of the query sequence. The next best hits are putative proteins, mainly annotated as type A sorting domain-containing proteins, from Formosa sp., where the percentage of identity is around 61%, but with an average coverage of the Fhf1 sequence much lower, around 74%.

**Figure 2:**
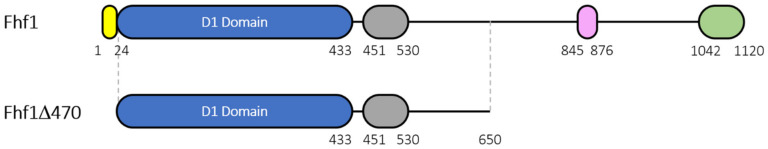
Primary structure and predicted domains of Fhf1 and Fhf1Δ470. Numbering corresponds to the full-length enzyme sequence. Domains are indicated as follows: yellow, peptide signal; blue, D1 N-terminal catalytic domain; grey, cadherin-like superfamily domain; pink, SprB repeat; green, secretion system C-terminal sorting domain.

InterProScan analysis [34] revealed several predicted conserved domains or repeats in the Fhf1 protein sequence. Hence, a cadherin-like superfamily domain (IPR015919) was predicted from position 451 to 530 (Figure 2). The fold consists of an Ig-like beta-sandwich with a Greek key topology [35]. The superfamily domain comprising the Ig-like domain is quite common in the GH107 family and has notably been found in the sequence of MfFcnA as three repeated domains [27], as well as in three endo-fucoidanases, Fp273, Fp277, and Fp279, recently isolated from a marine metagenome [36]. This Ig-like domain is generally associated with a calcium atom at the apical end [30]. However, the function of these domains in fucoidanases remains unclear and is still under investigation. Hence, the Ig-like domain does not appear to take part in any catalytic activity of the GH107 enzyme, but could play a role in fucoidan binding. 

An SprB repeat was predicted from position 845 to 876 (IPR025667) (Figure 2). SprB is described as a cell surface protein involved in gliding motility in the bacterium *Flavobacterium johnsoniae* [37]. At the C-terminal end of the protein, a secretion system C-terminal sorting domain (IPR026444) is identified between positions 1042 and 1120 (Figure 2). This domain is also predicted in previously described bacteroidetes fucoidanases, notably MfFcnA, FFA1, FFA2, AXE80_07420, and AXE80_07305 [30]. The N-terminal catalytic domain, named D1, is predicted between positions 25 to 433 in Fhf1 (Figure 2) based on sequence alignment with MfFcnA whose 3D structure was recently solved [30].

### 2.2. Sequence Comparisons of Fhf1 to Other Fucoidanases 

Fhf1 was compared with other known fucoidanase sequences of different specificities, namely, the endo α-(1,4) fucoidanases MfFcnA from *M. fucanivorans* (NCBI CAI47003.1, [27]) and FFA2 from *F. algae* (NCBI WP_057784219.1, [29]) and with enzymes known to hydrolyze α-(1,3) fucosyl bonds in fucoidans, namely, Fda1 and Fda2 from *Alteromonas* sp. (NCBI AAO00508.1 and AAO00509.1, respectively [26]). SVI_0379 from *Shewanella violacea* DSS12 was also included in the sequence comparison analysis, but the specificity of this enzyme has not yet been elucidated.

The similarity of the catalytic D1 domain (the most conserved domain among the classified members of GH107) between Fhf1 and other GH107 fucoidanases was compared by BLASTp analysis (Table 1). The D1 catalytic domain of Fhf1 shares the highest sequence identity with the catalytic domains of α-(1,4) fucoidanases MfFcnA (69% identity) and FFA2 (64%). The identity shared with the α-(1,3) fucoidanases Fda1 and Fda2, was much lower (24%) (Table 1).

Selected conserved motifs of Fhf1 and the known GH107 fucoidanases were aligned. We particularly focused on the conserved motifs surrounding the catalytic amino acids and the amino acids of the −1 subsite, identified in the crystal structure of MfFcnA and in a recently reported fucoidanase P5AFcnA from a marine *Psychromonas* sp. [30]. The catalytic aspartate playing the role of the nucleophile and the histidine, acting as the acid base catalyst, are identified at positions 225 and 293 in the Fhf1 protein sequence, respectively (Table 2). The four amino acids constituting the –1 subsite are identified in the Fhf1 sequence as Y144, N146, N268, and W366 (Table 3). Interestingly, all amino acids are conserved among the six GH107 enzymes, except at position 146 in Fhf1, which is an alanine for the α-(1,3) acting fucoidanases (Fda1 and Fda2) and asparagine for the characterized α-(1,4) fucoidanases and Fhf1 (Table 3, motif 1).

### 2.3. Construction of a Truncated Version and Recombinant Expression and Purification of Fhf1

The recombinant Fhf1 protein was constructed without the 24 amino acid signal peptide and with a C-terminal His tag to give a predicted molecular weight of 121 kDa. The Fhf1 protein was co-expressed in *E. coli* BL21 (DE3) with the pGro7 chaperone. Despite several attempts, the purification of the full length protein did not give satisfying results, possibly due to degradation of the protein during expression in *E. coli* (Figure S1). 

A truncated version of Fhf1, called Fhf1Δ470, and lacking the last 470 amino acids at the C-terminal end, was then constructed to improve the recombinant production and subsequent affinity chromatography purification. The C-terminal deletion was designed from sequence alignment with a FcnA C-terminal deletion mutant, FcnAΔ229. This mutant has been designed in our lab and was found to retain activity and to express and purify well [38]. For Fhf1Δ470, the 470 amino acid long C-terminal deletion meant that both the SprB repeat and the secretion system C-terminal sorting domain were deleted, while the Ig-like domain was retained (Figure 2). Fhf1Δ470 is 637 amino acids long and has a predicted molecular weight of 71 kDa. Fhf1Δ470 was expressed, and upon purification, the protein gave a band at the expected size of 71 kDa in both SDS-PAGE and western blot analysis (Figure S1). 

Fhf1 and Fhf1Δ470 were both active on fucoidan isolated from *F. evanescens* and showed comparable specificity, as demonstrated by the production of fuco-oligosaccharides of the same size by Carbohydrate PolyAcrylamide Gel Electrophoresis (C-PAGE) (Figure S2). This indicates that the C-terminal deletion does not affect the catalytic properties of the enzyme. All further analyses were then performed with Fhf1Δ470. 

### 2.4. Substrate Specificity of the Fhf1Δ470 Fucoidanase

The substrate specificity of Fhf1Δ470 was investigated using several different fucoidan substrates of varying structure and type of linkages, including fucoidans from *Undaria pinnatifida* and *Saccharina cichorioides* and linked only by α-(1,3) linkages (class 1 fucoidans) and fucoidans extracted from *F. evanescens* and *Fucus vesiculosus* with fucosyl residues alternatively linked through α-(1,3) and α-(1,4) glycosydic bonds (class 2 fucoidans). Additionally, galactofucans from *Sargassum mcclurei* and *Turbinaria ornata* were tested. 

**Figure 3:**
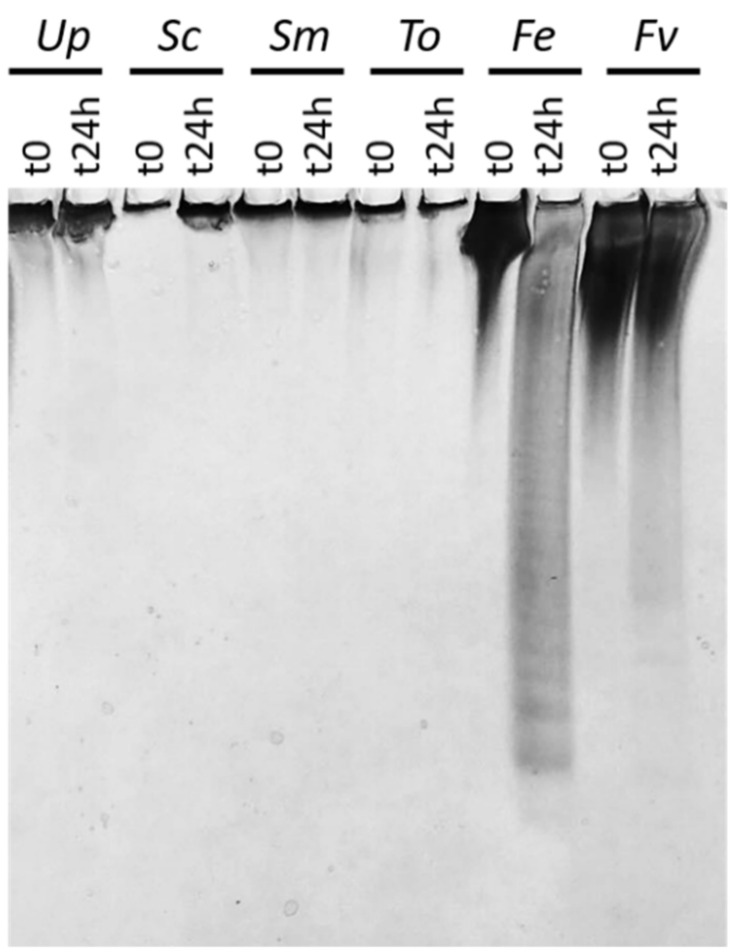
Substrate specificity of Fhf1Δ470. Reaction on different fucoidan substrates from various brown seaweed sources at *t* = 0 and after 24 h of reaction at pH 8 and 37 °C, analyzed by C-PAGE. Indices: fucoidan extracted from Up: *Undaria pinnatifida*; Sc: *Saccharina cichorioides*; Fe: *Fucus evanescens*; Fv: *Fucus vesiculosus* and galactofucans extracted from Sm: *Sargassum mcclurei*; To: *Turbinaria ornata*.

Fucoidanase activity was assessed using C-PAGE of the reaction products after 24 h reaction at pH 8, 37 °C. Fhf1Δ470 showed the highest activity on α-(1,3)/α-(1,4) fucoidan from *F. evanescens*, where fucosyl residues are mainly 2-O-sulfated (a smaller proportion is 2,4-di-O-sulfated) [39,40]. Fhf1Δ470 displayed lower activity on fucoidan isolated from *F. vesiculosus* (Figure 3). We interpret the lower activity is due to the nature of the fucoidan substitutions; although fucosyl residues in fucoidan from *F. vesiculosus* are mainly 2-O-sulfated, this substrate has a higher sulfation degree than the fucoidan from *F. evanescens* and a small proportion of 2,3-di-O-sulfated and 2,4-di-O-sulfated fucosyls [41]. The data show that Fhf1Δ470 catalyzes hydrolysis of class 2 fucoidans extracted from *F. evanescens* and *F. vesiculosus*, in which the fucosyl residues are alternatively linked through α-(1,3) and α-(1,4). The enzyme seems to target α-(1,4) linked fucosyl residues as it displayed no activity on the tested fucoidans with α-(1,3) linked fucosyl backbones (Figure 3). The preference of the enzyme for α-(1,3)/α-(1,4) fucoidans was consistent with the sequence analysis, which showed that the Fhf1 enzyme is related to other endo α-(1,4) fucoidanases of family GH107. 

### 2.5. Biochemical Characterization of the Fhf1Δ470 Fucoidanase

**Figure 4:**
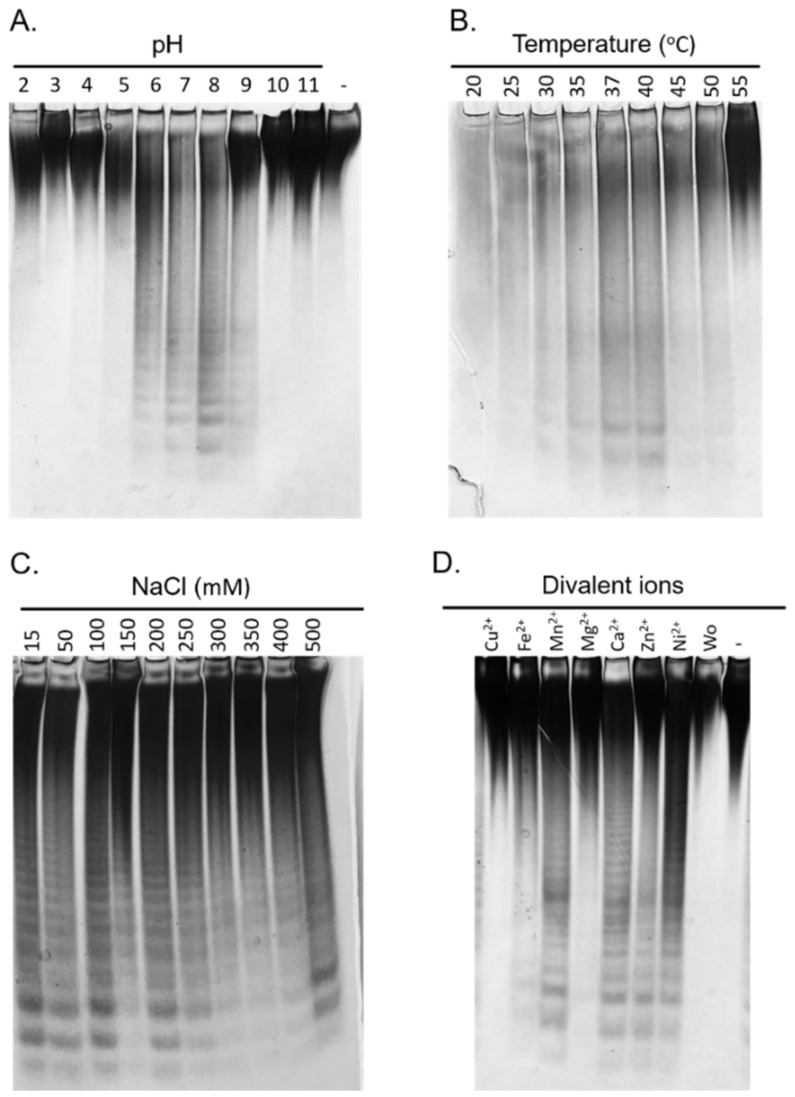
Biochemical properties of Fhf1Δ470 are illustrated by C-PAGE analysis. (**A**) pH optimum. The activity of Fhf1Δ470 was determined at different pH values ranging from 2 to 11. The minus symbol indicates the negative control where no enzyme was added. (**B**) Temperature optimum. Fhf1Δ470 activity was determined at different temperatures ranging from 20 to 55 °C. (**C**) Effect of NaCl. The tolerance to NaCl was investigated for concentrations from 15 to 500 mM. (**D**) Effect of different divalent cations. Wo indicates no ions added to the reaction after EDTA treatment; the minus symbol indicates the negative control where no enzyme was added. All reactions were run for 90 min using 10 g·L^−1^ of fucoidan extracted from *F. evanescens* and 0.1 mg·mL^−1^ of enzyme.

The biochemical properties of the Fhf1Δ470 enzyme were investigated using fucoidan extracted from *F. evanescens* as substrate. This substrate was extracted and purified from the seaweed using an enzyme-assisted methodology, that preserves the integrity of the polysaccharide [42]. Fhf1Δ470 displayed fucoidanase activity between pH 5 and pH 9, with an apparent optimum at pH 8 and was active at a wide range of temperatures from 20 to 50 °C, with an optimum around 37–40 °C (Figure 4A,B). These optimal working conditions are typical for marine bacterial fucoidanases [21]. 

The enzyme tolerated a wide range of NaCl concentrations from 15 to 500 mM (Figure 4C), indicating that the concentration of NaCl appeared to have no major effects on the activity of Fhf1Δ470. The effect of different divalent cations on the activity of the Fhf1Δ470 fucoidanase was also investigated. After EDTA treatment, i.e., when depleted of divalent cations, Fhf1Δ470 showed no activity (Figure 4D). However, when each of the ions Ca^2+^, Zn^2+^, Mn^2+^, and Ni^2+^ were added, the activity of the enzyme was restored (Figure 4D). While Fe^2+^ slightly activated the enzyme, the presence of Cu^2+^ and Mg^2+^ did not result in a significant increase in the activity. Those results are consistent with previous data showing that GH107 enzymes generally need Ca^2+^ ions to display activity on fucoidans [27,43]. This result agrees with the findings that Ca^2+^ binding sites have been identified in the crystal structures of both MfFcnA and P5FcnA [30].

### 2.6. Time-Course Hydrolysis of *F. evanescens* Fucoidan Using Fhf1Δ470 

**Figure 5:**
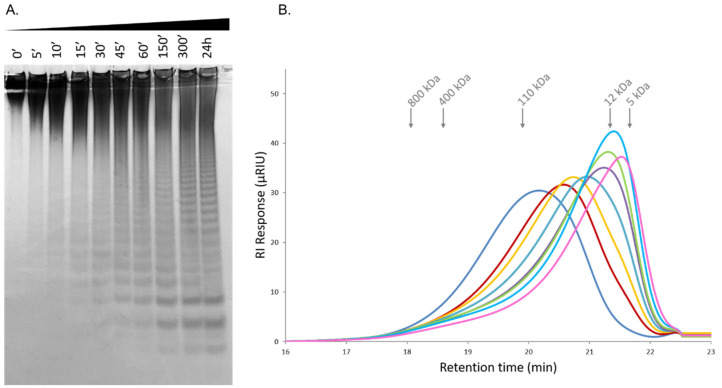
Kinetics of degradation of *F. evanescens* fucoidan catalyzed by Fhf1Δ470 fucoidanase; time-course of reaction at pH 8 and 37 °C. (**A**) C-PAGE electrophoresis, with minutes of reaction, indicated as 0’, 5’, 10’ etc. (**B**) HPSEC-RI chromatograms. The average molecular weights of the pullulan standards are indicated by grey arrows. Chromatogram shown in different colors corresponds to sample analysis after different times of reaction: from left to right: dark blue: *t* = 0, red: *t* = 15 min, yellow: *t* = 30 min, orange: *t* = 45 min, dark green: *t* = 60 min, light green: *t* = 150 min, light blue: *t* = 300 min; pink: *t* = 24 h.

The degradation of the fucoidan from *F. evanescens* by the fucoidanase Fhf1Δ470 under optimal reaction conditions, was monitored over 24 h and analyzed by C-PAGE and size-exclusion chromatography analysis coupled to a refractive index detector (HPSEC-RI). The degradation of *F. evanescens* fucoidan started after 15 min and was the highest after 24 h of reaction. Low molecular weight fuco-oligosaccharides were detected after 60 min of reaction (Figure 5A). Over time, the average molecular weight of the products decreased from ~80 kDa to 6.4 kDa, according to the HPSEC-RI analysis, indicating an endolytic mode of action of the enzyme (Figure 5B). 

### 2.7. Structural Determination of the Fucoidanase Hydrolysis Products

To establish the mode of action and detailed substrate specificity of Fhf1Δ470, the released products from the enzymatic hydrolysis of deacetylated fucoidan from *F. evanescens* were first separated into two fractions by ethanol precipitation: low molecular weight fuco-oligosaccharides products (LMP) and high molecular weight products (HMP) (Figure S3). The LMP were further separated using anion-exchange chromatography on Q-Sepharose HP to give three different pure fractions named Fr1, Fr2, and Fr3 (Figure S4). The yield of purified fractions Fr1, Fr2, and Fr3 were 2.9 mg (0.58%), 4.9 mg (0.98%) and 4.1 mg (0.82%), respectively.

The structures of the HMP and the three purified fractions Fr1, Fr2, and Fr3 were investigated by NMR spectroscopy using 1D and 2D techniques (1H, 13C, COSY, TOCSY, ROESY, HMBC).

**Figure 6:**
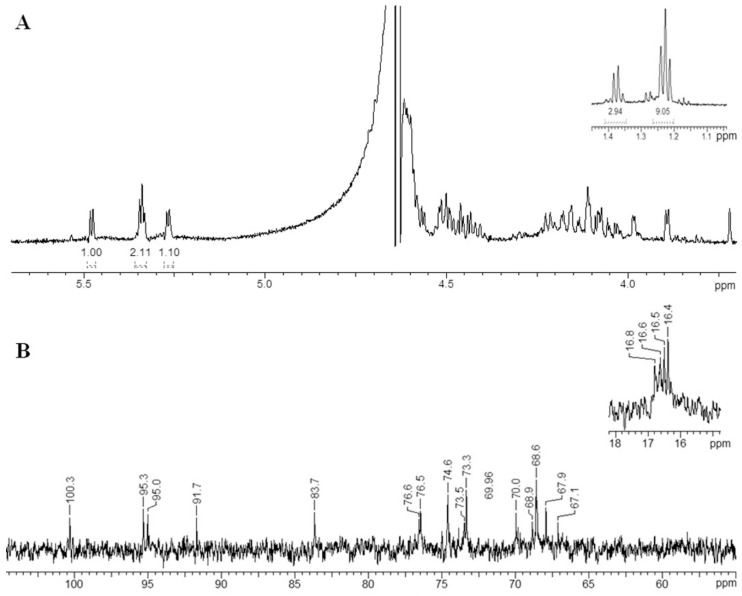
^1^H spectrum (**A**) and ^13^C spectrum (**B**) of Fr1 oligosaccharide.

The ^1^H and ^13^C NMR spectra of the Fr1 fraction (Figure 6) showed four signals with the same integrated intensities in the anomeric region, which indicated the presence of a tetrasaccharide carbohydrate backbone. The signals within each spin system were assigned using a ^1^H, ^1^H-COSY, ^1^H, ^1^H-TOCSY and ^1^H,^13^C-HSQC experiments (Table 4), and the identity of α-l-Fucp residues (units **A_1_**, **B_1_**, **C_1_**, and **D_1_**), was established by the characteristic coupling patterns and ^3^J_H,H_ constant values. Linkage and sequence analyses of the Fr1 were performed using a ^1^H,^1^H-ROESY, and a ^1^H,^13^C-HMBC experiments. The ^1^H,^1^H-ROESY spectrum revealed the following inter-residue cross-peaks showing the spatial proximity of the transglycosidic protons: H-1 **A_1_**/ H-4 **B_1_** at δ_Н_/δ_Н_ 5.27/3.99, H-1 **B_1_**/ H-3 **D_1_** at δ_Н_/δ_Н_ 5.34/4.05, and H-1 **C_1_**/ H-3 **A_1_** at δ_Н_/δ_Н_ 5.34/4.17. Accordingly, the ^1^H,^13^C-HMBC spectrum also displayed the following inter-residue cross-peaks: H-1 **A_1_**/ C-4 **B_1_** at δ_Н_/δ_C_ 5.27/83.7, H-1 **B_1_**/ C-3 **D_1_** at δ_Н_/δ_C_ 5.34/73.9, and H-1 **C_1_**/ C-3 **A_1_** at δ_Н_/δ_C_ 5.34/73.5. These data indicated that the Fr1 oligosaccharide has a linear structure and demonstrated that residue **A_1_** corresponded to a 3-substituted α-l-Fucp, residue **B_1_** to a 4-substituted α-l-Fucp, residue **D_1_** to a 3-substituted α-l-Fucp at the reducing end, and residue **C_1_** to a α-l-Fucp at the non-reducing end. The significant low-field positions of the signals for C-2 of all sugar residues (δ_C_ 74.6–76.6) when compared with their position in the non-substituted α- l-Fucp residue, indicated the locations of the sulfate groups.

**Figure 7:**
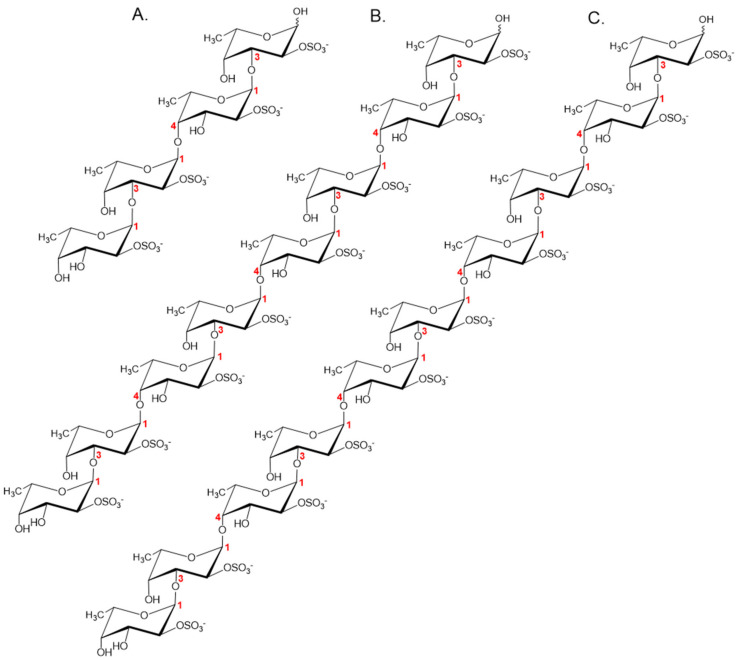
Structure of the fuco-oligosaccharides Fr1 (**A**), Fr2 (**B**), and Fr3 (**C**) released by Fhf1Δ470.

Based on the obtained data, the fraction Fr1 is a sulfated tetrasaccharide with the following structure: α-l-Fucp2S-(1→3)-α-l-Fucp2S-(1→4)-α-l-Fucp2S-(1→3)-α-l-Fucp2S (Figure 7A). Previously, an identical oligosaccharide was isolated from the low-molecular weight fucoidan hydrolysis products released by the fucoidanase FFA2 from *F. algae* [29].

Structures of fractions Fr2 and Fr3 were studied in a similar manner, as described for Fr1, and the assigned chemical shifts for these fractions are reported in Tables S1 and S2, respectively. The integrated intensity analysis of the protons in the anomeric regions of the ^1^H NMR spectra of Fr2 and Fr3 oligosaccharides showed the presence of an octasaccharide and a decasaccharide carbohydrate backbone, respectively (Figures S5 and S6). The linkage and sequence analyses of Fr2 and Fr3 were performed using ^1^H,^1^H-ROESY and ^1^H,^13^C-HMBC, and the location of sulfate groups was identified on the basis of known regularities of O-sulfation (α- and β-effects). 

Based on the obtained data, fractions Fr2 and Fr3 are octa- and decasaccharides with the structures α-l-Fucp2S-(1,3)-α-l-Fucp2S-(1,4)-α-l-Fucp2S-(1,3)-α-l-Fucp2S-(1,4)-α-l-Fucp2S-(1,3)-α-l-Fucp2S-(1,4)-α-l-Fucp2S-(1,3)-α-l-Fucp2S and α-l-Fucp2S-(1,3)-α-l-Fucp2S-(1,4)-α-l-Fucp2S-(1,3)-α-l-Fucp2S-(1,4)-α-l-Fucp2S-(1,3)-α-l-Fucp2S-(1,4)-α-l-Fucp2S-(1,3)-α-l-Fucp2S-(1,4)-α-l-Fucp2S-(1,3)-α-l-Fucp2S, respectively (Figure 7B,C). 

### 2.8. Proposed Fhf1 Mode of Action

**Figure 8:**
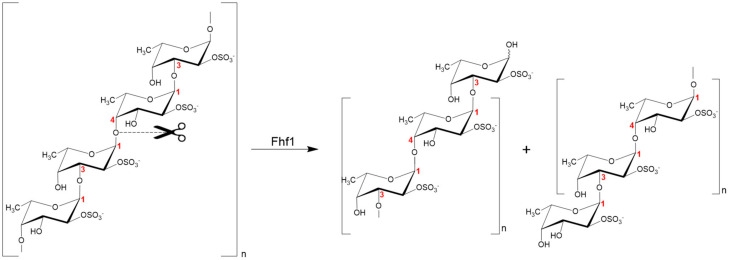
The proposed reaction catalyzed by the Fhf1Δ470 endo-fucoidanase.

Based on the elucidation of the structures of the fuco-oligosaccharides obtained by enzymatic hydrolysis, the fucoidanase Fhf1 is specific for the cleavage of α-(1,4)-glycosidic bonds between 2O-sulfated l-fucose residues in the structural motif [→3)-α-l-Fucp2S-(1,4)-α-l-Fucp2S-(1→] of fucoidan isolated from *F. evanescens* (Figure 8). Similar substrate specificity was reported for the fucoidanase FFA2 from *F. algae* [29]. Nevertheless, in comparison to FFA2, Fhf1, mainly produces oligosaccharides with a higher degree of polymerization (DP = 8–10 compared to DP = 4 in FFA2), which may indicate differences between Fhf1 and FFA2 in the topology of their active sites. It is likely that Fhf1 is inhibited by the final reaction products (di- and/or tetrasaccharides), which could explain why the oligosaccharides were not degraded further.

## 3. Materials and Methods 

### 3.1. Fucoidan Substrates

*S. mcclurei* and *T. ornata* seaweeds were collected in NhaTrang Bay, Vietnam, by trained staff from the NhaTrang Institute of Technology Research and Application. Crude fucoidans were extracted from seaweeds, as previously described [44]. Fucoidan from *S. mcclurei* was further purified by ion-exchange chromatography. *F. evanescens* from Kiel fjord, Germany, was collected in March 2017. The enzymatic extraction and purification of fucoidan from *F. evanescens* was performed as previously described [42]. The *S. cichorioides*, *F. evanescens* (for NMR analysis), and *U. pinnatifida* seaweeds were collected from Troitsa Bay, Sea of Japan, Far East of Russia (*S. cichorioides*, September 2015, *U. pinnatifida*, July 2015, *F. evanescens*, July 2017). Fresh seaweeds were powdered and pretreated with 70% aqueous ethanol (w/v = 1:10) for 10 days. The defatted seaweeds were air-dried at room temperature. Crude fucoidans from *S. cichorioides* and *U. pinnatifida* were obtained as previously described [44] and purified by ion-exchange chromatography [45]. For NMR experiments, *F. evanescens* fucoidan collected from Troitsa Bay was deacetylated as follows. Fucoidan (1 g) was first dissolved in 100 mL of 12 % NH_4_OH at 37 °C, overnight. Fucoidan solution was then dialyzed against 10 L of water for 48 h at 4 °C using SnakeSkin™ Dialysis Tubing (10K MWCO, 22 mm, Thermo Scientific). The solution of the deacetylated fucoidan obtained was then lyophilized.

Pure fucoidan from *F. vesiculosus* was purchased from Sigma-Aldrich (Steinheim, Germany).

### 3.2. Identification of the Fhf1 Gene and Sequence Analysis 

The fhf1 gene was identified in the genome of *F. haliotis* (Genbank accession number BDEL00000000, [32]) by performing a nucleotide BLAST against a small set of fucoidanase-encoding genes (GH107 endo-fucoidanase encoding genes). The protein sequence of Fhf1 was deposited under the NCBI accession number WP_066217780.1. Domains of Fhf1 were predicted using InterPro (https://www.ebi.ac.uk/interpro/), and the signal peptide was predicted using the SignalP 5.0 server (http://www.cbs.dtu.dk/services/SignalP/). 

Protein sequences used for comparison with Fhf1 were MfFcnA, from *M. fucanivorans* (Genbank accession number CAI47003.1), FFA2 from *F. algae* (genbank number WP_057784219.1), SVI_0379 from *S. violacea* DSS12 (Genbank accession number BAJ00350.1), Fda1 and Fda2 from *Alteromonas* sp. (genbank numbers AAO00508.1 and AAO00509.1, respectively). Protein sequence comparison was performed using either protein BLAST (NCBI) for local alignments or Clustal Omega (https://www.ebi.ac.uk/Tools/msa/clustalo/) for global alignments.

### 3.3. Cloning of the Fhf1 and Fhf1Δ470 Genes

The constructs containing the gene encoding Fhf1 and Fhf1Δ470 were designed to harbor a C-terminal 10xhistidine tag. The synthetic codon-optimized genes, optimized for *E. coli* expression and all devoid of their original signal peptides, were synthesized by GenScript (Piscataway, NJ, USA) and inserted in the pET-31b(+) vector between the NdeI and XhoI restriction sites.

E. coli DH5α (Thermo Fisher Scientific, Waltham, MA, USA) was used as plasmid propagation host. *E. coli* BL21 (DE3) cells (Thermo Fisher Scientific, Waltham, MA, USA) were used as an expression host. 

### 3.4. Production of Recombinant Enzymes 

Expression of Fhf1 and Fhf1Δ470 was performed in *E. coli* BL21 (DE3) harboring the Pch2 (pGro7) plasmid. Overnight cultures were grown at 37 °C under agitation (180 rpm) in lysogeny broth (LB) medium containing 100 μg·mL^−1^ ampicillin and 34 μg·mL^−1^ chloramphenicol were used to inoculate 500 mL LB supplemented with 100 μg·mL^−1^ ampicillin, 34 μg·mL^−1^ chloramphenicol, and 0.05% arabinose and incubated at 37 °C. When the OD_600_ of each culture reached a value between 0.6–0.8, gene expression was induced using 1 mM IPTG for 20 h at 20 °C, under agitation.

Cells were harvested by centrifugation (5000× *g* for 20 min), and the pellet was re-suspended in binding buffer (20 mM sodium phosphate buffer, 100 mM NaCl, 20 mM imidazole, pH 7.4) before sonication using a UP 400S Ultrasonic processor (Hielscher Ultrasonics, Germany). Cell debris was removed by centrifugation (20,000× *g*, 20 min at 4 °C), and the supernatant was filtered through a 0.45 μm filter and applied to a 5 mL Ni^2+^ Sepharose resin (GE Healthcare, Chicago, IL, USA), previously equilibrated with binding buffer. Proteins were eluted using a gradient of imidazole (20 to 500 mM imidazole in binding buffer). Imidazole was removed using PD-10 columns (GE Healthcare, Chicago, IL, USA), according to supplier’s recommendations. Proteins were visualized on SDS-PAGE and western blot. Protein concentration was determined using the Pierce™BCA protein assay kit (ThermoFisher Scientific, Waltham, MA, USA) with bovine serum albumin as standard.

### 3.5. SDS-PAGE Electrophoresis

The purity of the proteins was assessed by SDS–PAGE electrophoresis. Electrophoresis was performed in 12% precast acrylamide gels (Bio-Rad, Hercules, CA, USA) by adding 4xLaemmli loading-buffer (Bio-Rad, Hercules, CA, USA) supplemented with 5 mM of DTT to 5 µg purified protein. Gels were run in Tris-Glycine running buffer at 200 V for 35 min, and proteins were colored by Coomassie brilliant blue (Bio-rad, Hercules, CA, USA). Precision Plus Protein™ Unstained Protein Standard (Bio-Rad, Hercules, CA, USA) was used as standard. 

### 3.6. Western Blot Analysis 

Pure enzymes (5 µg) were first separated by SDS-PAGE electrophoresis using 12% acrylamide gel (Bio-Rad, Hercules, CA, USA). Proteins were then transferred onto a PVDF blotting membrane (GE Healthcare, Chicago, IL, USA) using Tris-glycine pH 8.3 running buffer at 100 V for 45 min. After the transfer of the protein, the membrane was blocked with 2% skimmed milk in TBS buffer, pH 7.6 for 1 h. The membrane was then incubated for 1 h in TBS buffer supplemented with a 1:10.000 diluted monoclonal anti-polyHistidine peroxidase-conjugated antibody (Sigma-Aldrich, Steinheim, Germany). After washing the membrane several times in TBS buffer, antibodies were detected by horseradish peroxidase using the AEC Kit (Sigma-Aldrich, Steinheim, Germany) according to the manufacturer’s protocol.

### 3.7. Activity Assays

Substrate specificity experiments were performed using an initial substrate concentration of 10 g·L^−1^ fucoidan, 10 mM CaCl_2_, and 100 mg·L^−1^ purified Fhf1Δ470, at pH 8 and 37 °C for 24 h. Time-course experiments were performed similarly using 10 g·L^−1^ purified fucoidan from *F. evanescens*, 10 mM CaCl_2_, and 100 mg·L^−1^ purified Fhf1Δ470, at pH 8 and 37 °C. The pH optimum was determined using 10 g·L^−1^ *F. evanescens* fucoidan, 10 mM CaCl_2_, and 100 mg·L^−1^ enzyme at 37 °C in 20 mM citrate-phosphate buffer at pH values ranging from 2 to 11 for 90 min. Temperature experiment reactions were performed using 10 g·L^−1^ *F. evanescens* fucoidan, 10 mM CaCl_2_, and 100 mg·L^−1^ of enzyme, at pH 8, for temperatures ranging from 20 to 55 °C for 90 min. The effects of divalent cations were investigated by performing enzymatic reactions using 10 g·L^−1^ *F. evanescens* fucoidan, 100 mg·L^−1^ of enzyme, and 10 mM of selected divalent ions at pH 8, and 37 °C, for 90 min. The enzyme was first incubated with 1 mM EDTA for 10 min to exclude the possibility of any bound metals prior to the analysis and EDTA was then removed by dialysis. The effects of NaCl concentrations were determined using 10 g·L^−1^ *F. evanescens* fucoidan, 10 mM CaCl_2_, and 100 mg·L^−1^ of enzyme, at pH 8, 37 °C for 90 min. All reactions were stopped by heating at 80 °C for 10 min. Samples were stored at −20 °C until further analyses. 

### 3.8. Carbohydrate–Polyacrylamide Gel Electrophoresis (C-PAGE) 

One volume of the reaction sample was mixed with one volume of loading buffer (20% glycerol and 0.02% phenol red). Samples (10 μL) were loaded on a 20% (w/v) 1 mm thick polyacrylamide gel and run at 400 V for 1.5 h, using 100 mM Tris-borate buffer pH 8.3 as running buffer. The gel was first stained with a solution containing 0.05% alcian blue 8GX (Panreac Quimica, Barcelona, Spain) in 2% acetic acid for 45 min and then with 0.01% O-toluidine blue (Sigma-Aldrich, Steinheim, Germany) in 50% aqueous ethanol and 1% acid acetic for 30 min. The gel was destained in water.

### 3.9. HPSEC-RI Analysis

High Performance Size Exclusion Chromatography was performed using an Ultimate iso-3100 SD pump with a WPS-3000 sampler (Dionex, Sunnyvale, CA, USA) connected to an RI-101 Shodex refractive index detector (Showa Denko K.K, Tokyo, Japan). 100 µL of reaction samples diluted three times was loaded on a Shodex SB-806 HQ GPC column (300 × 8 mm) equipped with a Shodex SB-G guard column (50 × 6 mm) (Showa Denko K.K, Tokyo, Japan). Elution was performed using 100 mM sodium acetate pH 6 at a flow rate of 0.5 mL·min^−1^ at room temperature. Pullulan of 800, 400, 110, 12, and 5 kDa (Sigma-Aldrich, Steinheim, Germany) were used as standards.

### 3.10. Oligosaccharides Isolation

Fucoidan from *F. evanescens* (500 mg) was enzymatically hydrolyzed in optimal working conditions using 100 mg·L^−1^ Fhf1, and 10 mM CaCl_2_ in Tris-HCl buffer, at pH 8 and 37 °C. After 72 h, the reaction was stopped by heating at 80 °C for 10 min. The high molecular weight reaction products (HMP) were precipitated with ice-cold ethanol at a ratio of 1:3 (v/v) and separated by centrifugation at 10,000× *g* for 15 min. The supernatant containing low molecular weight reaction products (LMP) was concentrated under vacuum and applied to a Q-Sepharose HP column (1 × 10 cm) (GE Healthcare, Chicago, IL, USA) equilibrated with water. Oligosaccharides were eluted with sequential linear gradients from 0–0.75 M, from 0.5–1.5 M, and from 1–2 M NH_4_HCO_3_, at a flow rate of 1 mL·min^−1^. The fractions containing the carbohydrates were pooled, concentrated, and desalted by vacuum evaporation or Sephadex G-10 column. The carbohydrates in the fractions were detected using the phenol-sulphuric acid method [46]. Fractions containing carbohydrates were analyzed by C-PAGE electrophoresis, as described previously [47]. Another hydrolysis reaction using a fresh enzyme was performed on precipitated and re-solubilized HMP to ensure there was no further hydrolysis. 

### 3.11. Nuclear Magnetic Resonance Spectroscopy

Nuclear Magnetic Resonance spectra were recorded using an Avance DPX-700 NMR spectrometer (Bruker Biospin AG, Fällanden, Switzerland) and/or an Avance DPX-500 NMR spectrometer (Bruker, Hamburg, Germany). 1H, 13C, 1D TOCSY (total correlated spectroscopy) spectra and two-dimensional (2D) spectra (correlation spectroscopy COSY, rotating frame nuclear Overhauser effect spectroscopy ROESY, nuclear Overhauser effect spectroscopy NOESY, heteronuclear single-quantum correlation spectroscopy HSQC, heteronuclear multiple-bond correlation spectroscopy HMBC) were recorded for solutions of poly- and oligosaccharides in D_2_O at 35–40 °C. The concentrations of the samples ranged between 3–10 mg·mL^−1^. Unfortunately, the structure of the HMP fraction could not be determined by NMR spectroscopy, due to insufficient spectral resolution for detailed interpretation. 

## 4. Conclusions

This study aimed to expand the repertoire of GH107 enzymes. Our objective was twofold: To provide new fundamental knowledge on GH107 fucoidanases and to identify new enzyme tools for selective fucoidan depolymerization for the production of homogenous bioactive fucoidan molecules. The structural diversity of fucoidans provides an opportunity for discovering fucoidanases with various specificities. Using genome mining, three genes coding for putative fucoidanases were identified in the genome of the marine bacterium *F. haliotis*, known to degrade brown macroalgae. One of these genes, *fhf1*, was singled out and cloned and expressed in *E. coli*. The native protein, Fhf1, was unstable, however. Hence, based on alignment comparison to other fucoidanases, a more stable version was constructed with the catalytic domain retained. This truncated version, called Fhf1Δ470 was devoid of the last 470 amino acids at the C-terminal end. The Fhf1Δ470 protein was successfully expressed, purified, and characterized. According to substrate specificity studies, time-course experiments, and in-depth analysis of the structure of the degradation products catalyzed by the enzyme, Fhf1 acts as an endo-α-(1,4)-fucoidanase and catalyzes the hydrolysis of α-(1,4) glycosidic bonds between 2-*O*-sulfated l-fucose residues in alternating motif [→3)-α-l-Fucp2S-(1,4)-α-l-Fucp2S-(1→] of fucoidan. This result was supported by our bioinformatic analyses, where Fhf1 shares common signature motifs and a higher percentage of identity with characterized endo-α-1,4-fucoidanases. The discovery of this enzyme and its stabilization by extensive C-terminal truncation provides new knowledge on the mode of action and function of bacterial GH107 enzymes, which may pave the way for improved production of bioactive fucoidan-oligosaccharides with pharmaceutical application potential. 

## Figures and Tables

**Table 1:** Homology of the catalytic domain, D1 of Fhf1 with other GH107 fucoidanases. The percentage of identity and similarity between the sequences was calculated using BLASTp analysis.

Enzyme	Organism	Accession Number	Linkage Specificity	Homology with Fhf1 (D1 Domain)	
				% Identity	% Similarity
MfFcnA	*Mariniflexile fuconivorans*	CAI47003.1	α-(1,4)	69	79
FFA2	*Formosa algae*	WP_057784219.1	α-(1,4)	64	77
SVI_0379	*Shewanella violacea*	BAJ00350.1	n.d	25	42
Fda1	*Alteromonas* sp.	AAO00508.1	α-(1,3)	24	39
Fda2	*Alteromonas* sp.	AAO00509.1	α-(1,3)	24	38

**Table 2:** Alignment of selected conserved motifs around the catalytic amino acids between Fhf1 and other known GH107 enzymes. MfFcnA, from *Mariniflexile fucanivorans* (Genbank accession number CAI47003.1), FFA2 from *Formosa algae* (genbank number WP_057784219.1), SVI_0379 from *Shewanella violacea* DSS12 (Genbank accession number BAJ00350.1), Fda1 and Fda2 from *Alteromonas* sp. (genbank numbers AAO00508.1 and AAO00509.1, respectively). The aspartate (D) acting as the nucleophile is indicated in red, while the histidine (H) acting as the acid base catalyst is indicated in blue. Below the alignment, an * (asterisk) indicates positions in the alignment which have a fully conserved residue. A colon (“:”) indicates conservation between amino acids of strongly similar properties, and a period (“.”) indicates conservation between groups of amino acids of weakly similar properties. I and II indicates the investigated protein domains.

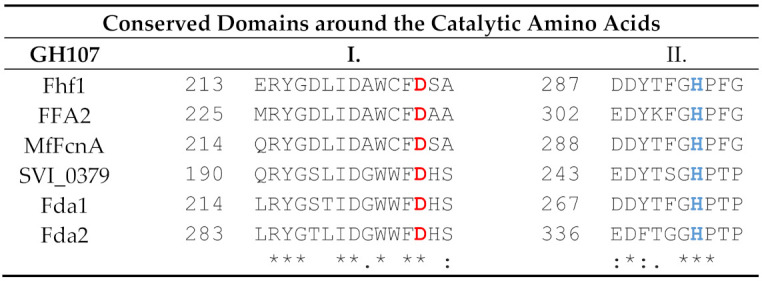

**Table 3:** Alignment of selected conserved motifs around the amino acids identified in the −1 subsite (indicated in purple) in the 3D structure of MfFcnA between Fhf1 and other known GH107 enzymes. MfFcnA, from *M. fucanivorans* (Genbank accession number CAI47003.1), FFA2 from *F. algae* (genbank number WP_057784219.1), SVI_0379 from *S. violacea* DSS12 (Genbank accession number BAJ00350.1), Fda1 and Fda2 from *Alteromonas* sp. (genbank numbers AAO00508.1 and AAO00509.1, respectively). Below the alignment, an * (asterisk) indicates positions in the alignment which have a fully conserved residue. A colon (“:”) indicates conservation between amino acids of strongly similar properties, and a period (“.”) indicates conservation between groups of amino acids of weakly similar properties. 1, 2 and 3 indicates the investigated protein domains.

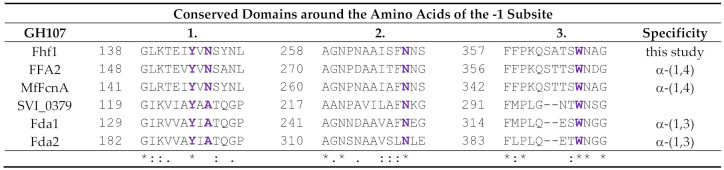

**Table 4:** ^1^H and ^13^C NMR data for the Fr1 oligosaccharide (δ, ppm).

Residue	H1/C1	H2/C2	H3/C3	H4/C4	H5/C5	H6/C6
**A_1_**	5.27/100.3	4.58/74.6	4.17/73.5	4.11/70.0	4.41/68.6	1.24/16.5
**B_1_**	5.34/95.3	4.47/76.6	4.15/68.6	3.99/83.7	4.51/68.9	1.38/16.8
**C_1_**	5.34/95.0	4.45/76.5	4.10/68.6	3.89/73.3	4.51/67.9	1.22/16.4
**D_1_**	5.48/91.7	4.51/74.6	4.05/73.9	4.07/69.8	4.22/67.1	1.24/16.6

## Supplementary Materials

The following are available online at https://www.mdpi.com/1660-3397/18/11/562/s1, Figure S1: Expression and purification of Fhf1 and Fhf1Δ470. A. SDS-PAGE of purified Fhf1; B. SDS-PAGE of purified Fhf1Δ470; C. Western Blot of purified Fhf1 and D. Western blot of purified Fhf1Δ470. M stands for protein ladder, and E for eluted fraction. Green arrows indicate the expected size of the purified proteins (121 KDa for Fhf1 and 71 kDa for Fhf1Δ470), Figure S2: Fucoidanase activity of Fhf1 and Fhf1Δ470 on fucoidan extracted from *Fucus evanescens* illustrated by C-PAGE. Both reactions have been performed at 37 °C, pH 8 with 10 mM CaCl_2_ 10 g·L^−1^ of fucoidan and using 0,1 mg·L^−1^ of enzyme for 90 min. Polysaccharides are retained at the top of the gel, while degradation products, e.g., oligosaccharides migrate down in the gel according to size and charge, Figure S3: Separation of high and low molecular weight fucoidans. A. released products from the enzymatic hydrolysis of the fucoidan from *F. evanescens* using Fhf1Δ470, LMP stands for low molecular weight products and HMP for high molecular weight products, separated by ethanol precipitation, Figure S4: Separation of LMP fraction obtained after enzymatic treatment of fucoidan from *F. evanescens* by the recombinant fucoidanase Ffh1Δ470. A. Elution profile of LMP fraction on Q-Sepharose HP column. B. C-PAGE analysis of eluted fractions. C. C-PAGE analysis of purified fractions Fr1, Fr2, Fr3 and LMP fraction, Figure S5: ^1^H spectrum (A) and ^13^C spectrum (B) of Fr2 fuco-oligosaccharide, Figure S6: ^1^H spectrum (A) and ^13^C spectrum (B) of Fr3 fuco-oligosaccharide.
