# Supplementary material for: Functional Characterization of a New GH107 Endo-α-(1,4)-Fucoidanase from the Marine Bacterium Formosa haliotis
Source: Mar Drugs. 2020 Nov 17;18(11):562. doi: 10.3390/md18110562 (PMC7698502; doi:10.3390/md18110562)
Supplement: Supplementary file 1 [file marinedrugs-18-00562-s001.pdf]

marine drugs

Supplementary material

Functional Characterization of a New GH107 Endo-α-(1,4)-Fucoidanase from the Marine Bacterium *Formosa haliotis*

Marlene Vuillemin [1], Artem S. Silchenko [3], Hang Thi Thuy Cao [2], Maxim S. Kokoulin [3], Vo Thi Dieu Trang [1,2], Jesper Holck [1], Svetlana P. Ermakova [3], Anne S. Meyer [1], Maria Dalgaard Mikkelsen [1,*]

1 Protein Chemistry and Enzyme Technology Section, DTU Bioengineering, Department of Biotechnology and Biomedicine, Technical University of Denmark, 2800 Kgs. Lyngby, Denmark; mavu@dtu.dk (M.V.); tvtd@dtu.dk (V.T.D.T.); jesho@dtu.dk (J.H.); asme@dtu.dk (A.S.M.)
2 NhaTrang Institute of Technology Research and Application, Vietnam Academy of Science and Technology, 02 Hung Vuong Street, Nhatrang 650000, Vietnam; caohang.nitra@gmail.com (H.T.T.C.)
3 G.B. Elyakov Pacific Institute of Bioorganic Chemistry, Far-Eastern Branch of the Russian Academy of Sciences, 159, Prospect 100-let Vladivostoku, Vladivostok 690022, Russia; artem.silchencko@yandex.ru (A.S.S.); maxchem@mail.ru (M.S.K); ermakova@piboc.dvo.ru (S.P.E)
* Correspondence: mdami@dtu.dk; Tel.: +45-45-25-61-92

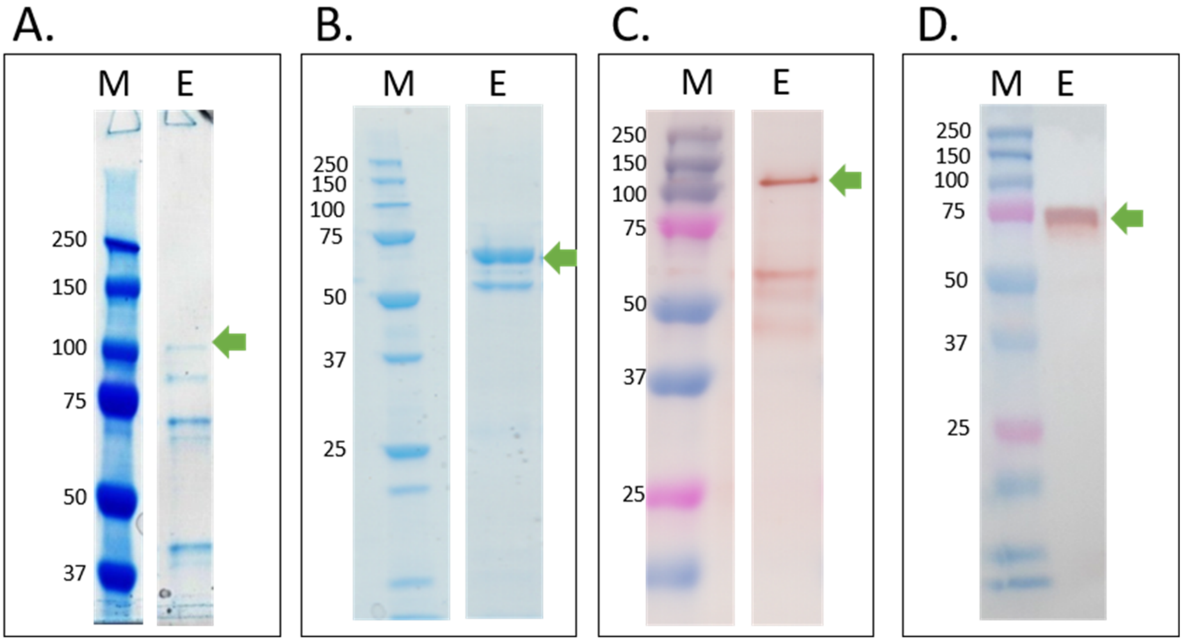

Figure S1. Expression and purification of Fhf1 and Fhf1Δ470. A. SDS-PAGE of purified Fhf1; B. SDS-PAGE of purified Fhf1Δ470; C. Western Blot of purified Fhf1 and D. Western blot of purified Fhf1Δ470. M stands for protein ladder, and E for eluted fraction. Green arrows indicate the expected size of the purified proteins (121 KDa for Fhf1 and 71 kDa for Fhf1Δ470).

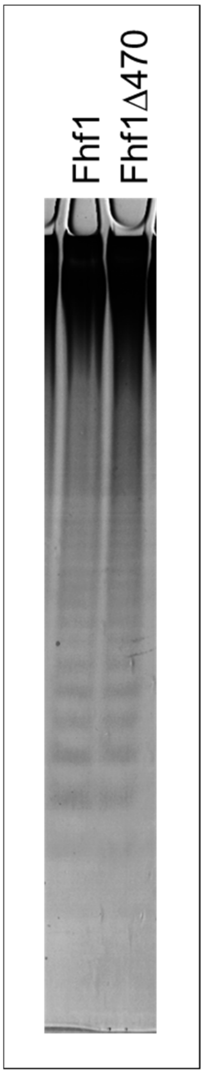

Figure S2. Fucoidanase activity of Fhf1 and Fhf1Δ470 on fucoidan extracted from *Fucus evanescens* illustrated by C-PAGE. Both reactions have been performed at 37 °C, pH 8 with 10 mM $CaCl_2$ 10 g·L^{-1} of fucoidan and using 0,1 mg·L^{-1} of enzyme for 90 minutes. Polysaccharides are retained at the top of the gel, while degradation products, *e.g.* oligosaccharides migrate down in the gel according to size and charge.

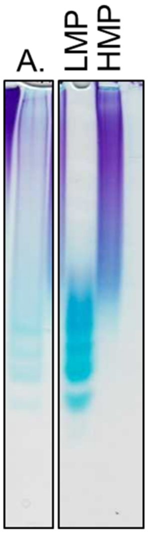

Figure S3. Separation of high and low molecular weight fucoidans. A. released products from the enzymatic hydrolysis of the fucoidan from *F. evanescens* using Fhf1Δ470, LMP stands for low molecular weight products and HMP for high molecular weight products, separated by ethanol precipitation.

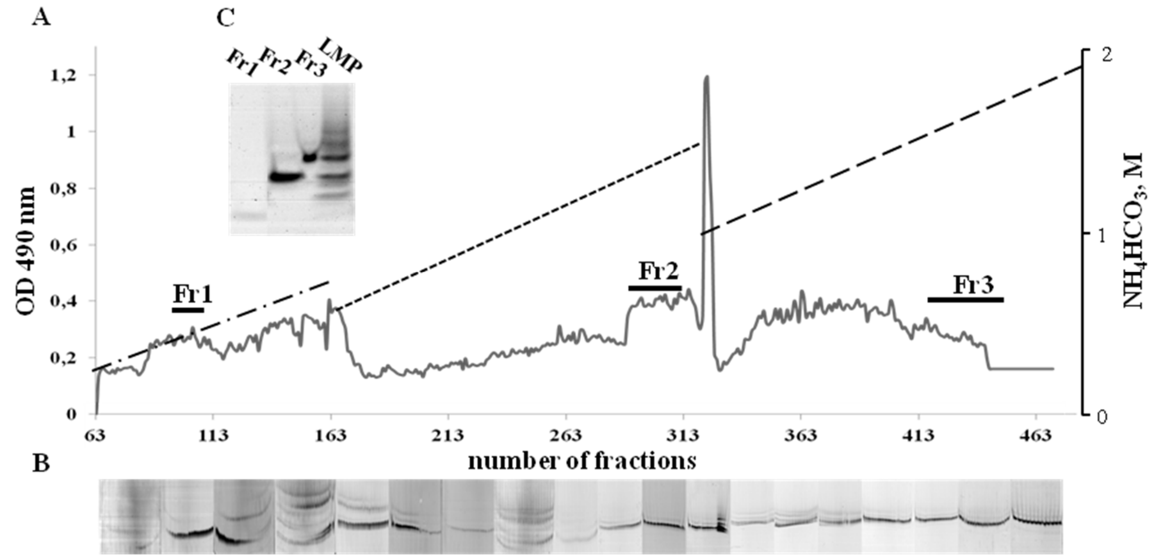

Figure S4. Separation of LMP fraction obtained after enzymatic treatment of fucoidan from *F. evanescens* by the recombinant fucoidanase Ffh1Δ470. A. Elution profile of LMP fraction on Q-Sepharose HP column. B. C-PAGE analysis of eluted fractions. C. C-PAGE analysis of purified fractions Fr1, Fr2, Fr3 and LMP fraction.

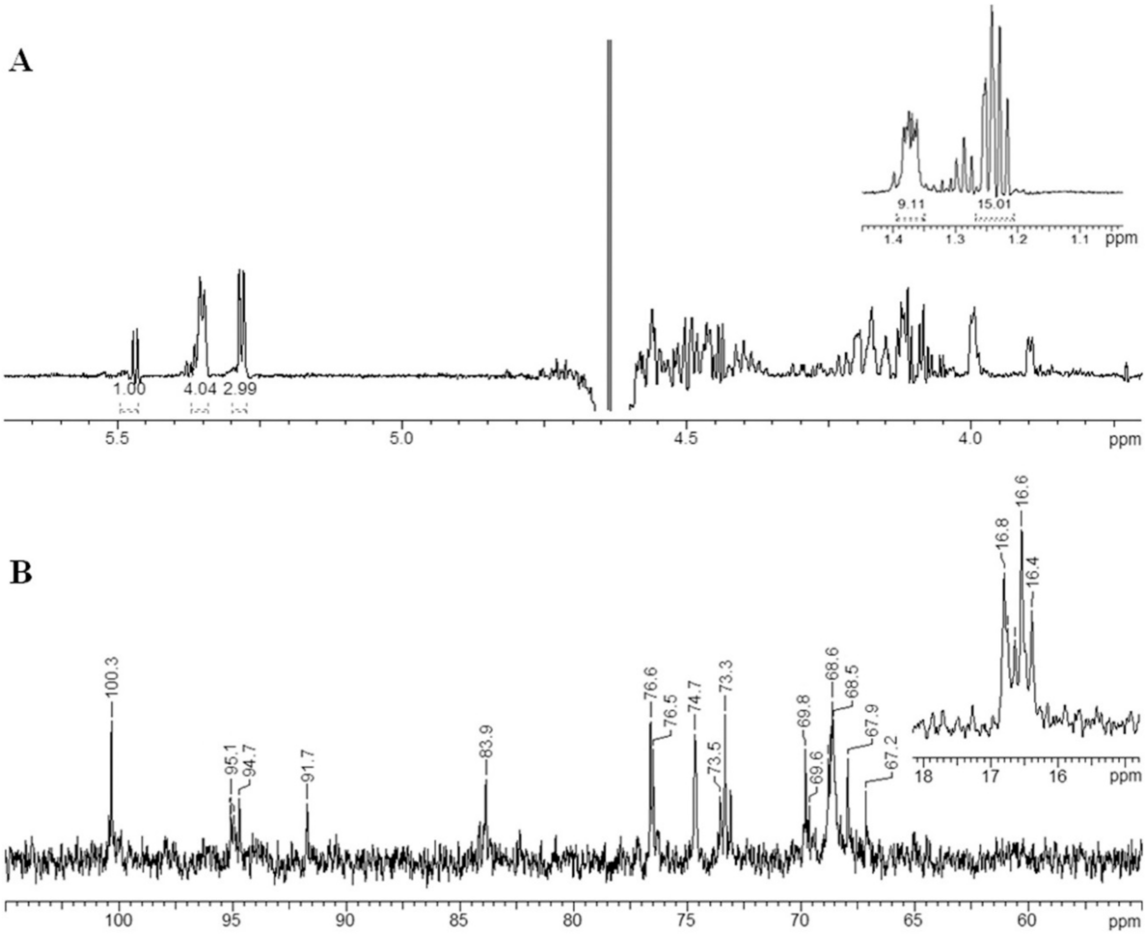

Figure S5. ^{1}H spectrum (**A**) and ^{13}C spectrum (**B**) of Fr2 fuco-oligosaccharide.

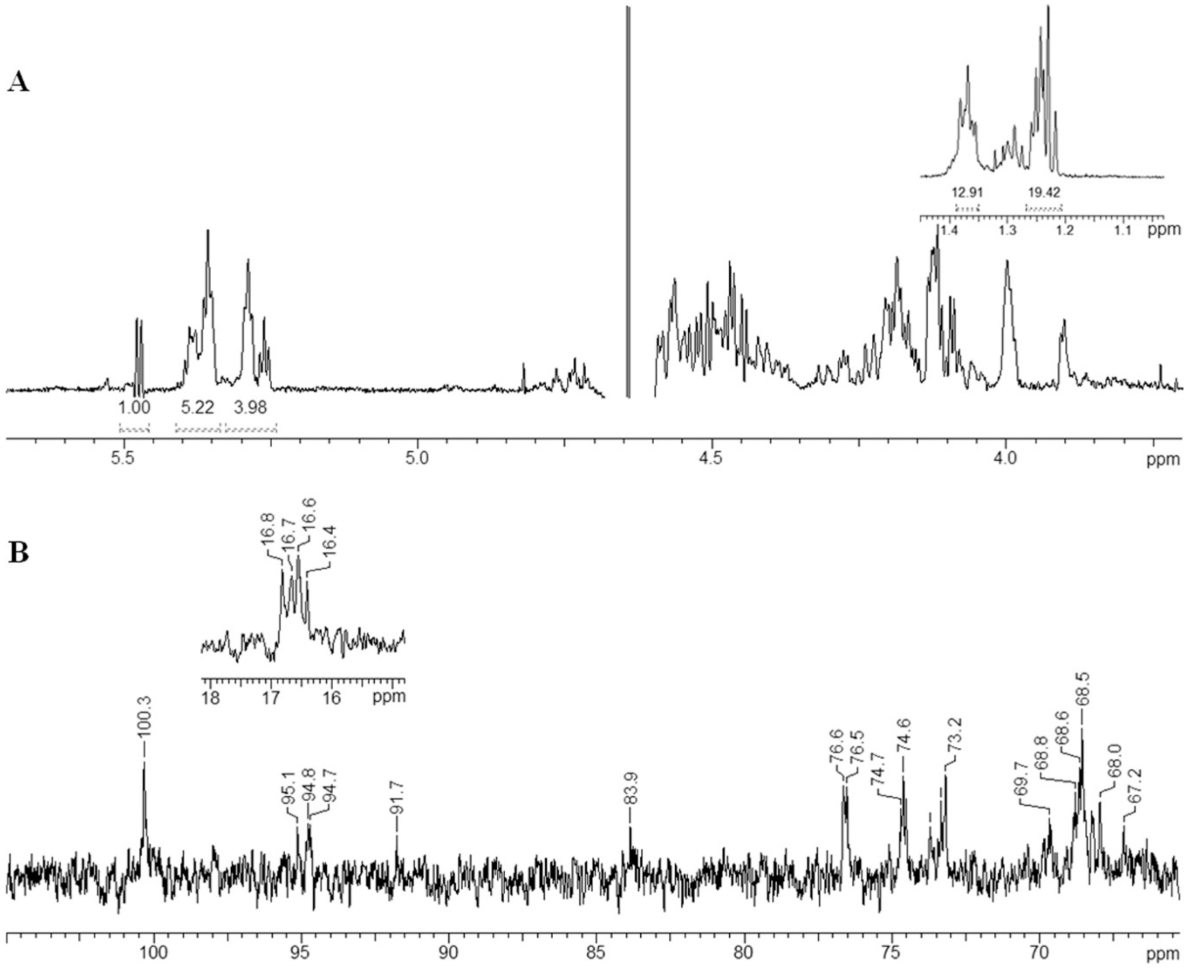

Figure S6. ^{1}H spectrum (**A**) and ^{13}C spectrum (**B**) of Fr3 fuco-oligosaccharide.

Table 1. ^{1}H and ^{13}C NMR data for the Fr2 oligosaccharide, (δ, ppm).

Residue	H1/C1	H2/C2	H3/C3	H4/C4	H5/C5	H6/C6
**A_2**	5.28/100.3	4.57/74.6	4.19/73.3	4.12/69.8	4.41/68.5	1.25/16.6
**B_2**	5.28/100.3	4.57/74.6	4.19/73.3	4.12/69.8	4.41/68.5	1.25/16.6
**C_2**	5.28/100.3	4.57/74.6	4.19/73.3	4.12/69.8	4.41/68.5	1.25/16.6
**D_2**	5.36/95.1	4.47/76.5	4.16/68.6	4.00/83.9	4.55/68.8	1.37/16.8
**E_2**	5.36/95.1	4.47/76.5	4.16/68.6	4.00/83.9	4.55/68.8	1.37/16.8
**F_2**	5.36/95.1	4.47/76.5	4.16/68.6	4.00/83.9	4.55/68.8	1.37/16.8
**G_2**	5.35/94.7	4.45/76.6	4.10/68.7	3.90/73.3	4.50/67.9	1.22/16.4
**H_2**	5.47/91.7	4.51/74.7	4.06/73.5	4.09/69.6	4.22/67.2	1.24/16.7

Table 2. ^{1}H and ^{13}C NMR data for the Fr3 oligosaccharide, (δ, ppm).

Residue	H1/C1	H2/C2	H3/C3	H4/C4	H5/C5	H6/C6
**A_3**	5.25/100.3	4.58/74.6	4.18/73.2	4.12/69.7	4.41/68.5	1.24/16.6
**B_3**	5.28/100.3	4.58/74.6	4.19/73.2	4.12/69.7	4.41/68.5	1.24/16.6
**C_3**	5.28/100.3	4.58/74.6	4.19/73.2	4.12/69.7	4.41/68.5	1.24/16.6
**D_3**	5.28/100.3	4.58/74.6	4.19/73.2	4.12/69.7	4.41/68.5	1.24/16.6
**E_3**	5.35/95.1	4.47/76.5	4.16/68.6	3.99/83.9	4.55/68.8	1.37/16.8
**F_3**	5.35/95.1	4.47/76.5	4.16/68.6	3.99/83.9	4.55/68.8	1.37/16.8
**G_3**	5.35/95.1	4.47/76.5	4.16/68.6	3.99/83.9	4.55/68.8	1.37/16.8
**H_3**	5.38/94.7	4.48/76.5	4.19/68.6	3.98/83.9	4.55/68.8	1.37/16.8
**I_3**	5.35/94.8	4.46/76.6	4.10/68.6	3.90/73.3	4.50/68.0	1.22/16.4
**J_3**	5.47/91.7	4.51/74.7	4.05/73.5	4.08/69.6	4.23/67.2	1.24/16.7
